# Mechanism-Based FE Simulation of Tool Wear in Diamond Drilling of SiC_p_/Al Composites

**DOI:** 10.3390/ma11020252

**Published:** 2018-02-07

**Authors:** Junfeng Xiang, Siqin Pang, Lijing Xie, Feinong Gao, Xin Hu, Jie Yi, Fang Hu

**Affiliations:** 1 School of Mechanical Engineering, Beijing Institute of Technology, Beijing 100081, China; xiang_junfeng@126.com (J.X.); pangsq@yeah.net (S.P.); 2120170328@bit.edu.cn (F.G.); 13810438099@163.com (X.H.); 3120140175@bit.edu.cn (J.Y.); songyl1919@163.com (F.H.); 2 Key Laboratory of Advanced Machining, Beijing Institute of Technology, Beijing 100081, China

**Keywords:** diamond tool, drilling, abrasive-chemical wear, graphitization, finite element, SiC_p_/Al6063 composites

## Abstract

The aim of this work is to analyze the micro mechanisms underlying the wear of macroscale tools during diamond machining of SiC_p_/Al6063 composites and to develop the mechanism-based diamond wear model in relation to the dominant wear behaviors. During drilling, high volume fraction SiC_p_/Al6063 composites containing Cu, the dominant wear mechanisms of diamond tool involve thermodynamically activated physicochemical wear due to diamond-graphite transformation catalyzed by Cu in air atmosphere and mechanically driven abrasive wear due to high-frequency scrape of hard SiC reinforcement on tool surface. An analytical diamond wear model, coupling Usui abrasive wear model and Arrhenius extended graphitization wear model was proposed and implemented through a user-defined subroutine for tool wear estimates. Tool wear estimate in diamond drilling of SiC_p_/Al6063 composites was achieved by incorporating the combined abrasive-chemical tool wear subroutine into the coupled thermomechanical FE model of 3D drilling. The developed drilling FE model for reproducing diamond tool wear was validated for feasibility and reliability by comparing numerically simulated tool wear morphology and experimentally observed results after drilling a hole using brazed polycrystalline diamond (PCD) and chemical vapor deposition (CVD) diamond coated tools. A fairly good agreement of experimental and simulated results in cutting forces, chip and tool wear morphologies demonstrates that the developed 3D drilling FE model, combined with a subroutine for diamond tool wear estimate can provide a more accurate analysis not only in cutting forces and chip shape but also in tool wear behavior during drilling SiC_p_/Al6063 composites. Once validated and calibrated, the developed diamond tool wear model in conjunction with other machining FE models can be easily extended to the investigation of tool wear evolution with various diamond tool geometries and other machining processes in cutting different workpiece materials.

## 1. Introduction

Silicon carbide particulate reinforced aluminum matrix (SiC_p_/Al) composites have been of great potential due to their superior physical and mechanical properties, such as high stiffness-to-weight ratio, high specific strength, high wear resistance, low sensitivity to temperature variations and excellent corrosion resistance [1]. This composite material has a promising application prospect in many advanced industries such as aerospace, marine, automotive and sport equipment [2,3]. However, SiC_p_/Al composites are limited in the actual production applications by their poor machinability since the hard SiC particles in the aluminum matrix lead to serious tool wear and undesired surface quality [4,5].

In cutting SiC_p_/Al composites, the conventional tools such as high-speed steel (HSS) and ceramics easily suffer from rapid tool wear, poor hole quality and higher cutting forces induced by tool wear [6,7,8]. Although the carbide tools are found to be superior machining performance to HSS and ceramics tools but fail to perform well in machining SiC_p_/Al composites with SiC volume fraction higher than 50% [9] due to severe tool wear. Hence, the diamond, diamond coated and diamond-like tools are considered one of the most favorable ones for cutting these materials [10,11]. 

Since progressive tool wear can weaken tool structure, cause obvious increase in cutting force and eventually lead to poor surface finish and catastrophic failure of cutting tools, the capability of predicting contribution of miscellaneous wear mechanisms to tool wear is very helpful for the selection of tool materials, redesign of tool geometry and optimization of cutting process [12]. Over the past several decades, the research upon tool wear has been mainly based on empirical method or experimental investigation [13,14]. Due to the excessive process parameters influencing tool wear, numerous cutting experiments need to be carried out to develop empirical relationships among them. Based on Taylor’s basic and extended equations, the empirical tool life models under very limited cutting conditions were developed. Many models based on extended Taylor’s tool life equation involving process variables were developed to describe more reliable tool life estimates and to extend its applicability to other cutting technologies. Colding [15] extended a generalized tool life estimate to a variety of machining technologies and derived the relationship between process parameters and tool life. Such relation has been modified further by Choudhury [16] that take into account tool geometry to achieve more accurate estimate for tool life, which in turn was used to maximize tool life subject to practical cutting conditions. However, although this empirical approach for tool life estimates aimed at some specific materials and tool geometries has gained a certain popularity, its applicability in solving general problems of tool wear is severely limited due to the lack of inherent physical meaning in these empirical life models. And only characterized by sufficient process parameters can these empirical models estimate tool life more accurately but the cost of tool wear experiments for identifying model parameters is often heavy. Moreover, except for the available tool life based on empirical Taylor model, it is impossible to get further information upon the tool wear progress, worn geometry, or even wear mechanism in different tool zones which are sometimes necessary and important for tool design and tool material selection [17]. Issues with empirical approach of tool life testing were identified. Regarding tool life testing, there are some ISO standard, e.g., ISO 8688-2 [18], which recommends testing procedure, tool wear modes, etc.

To establish the tool wear model with less process variables that can provide accurate tool life estimate and more wear details, many studies [19,20,21,22] were dedicated to developing the tool wear analytical models. These analytical models were characterized as the effects of internal variables such as the temperature at the tool-workpiece interface, stresses at the tool surface, relative sliding velocity between the tool and workpiece and microstructure of the tool and workpiece on tool wear in different wear modes involving abrasion, adhesion and diffusion. Among all analytical wear models, the wear one proposed by Takeyama [21] is used to describe the combined effect of mechanical abrasion proportional to sliding velocity and thermally activated diffusion in relation to interface temperature and activation energy for diffusion. Subsequently, the research of Usui [22], based on Shaw’ adhesive wear model [23], derived tool wear equation to incorporate the influence of interface temperature, relative sliding velocity, interface pressure upon tool wear rate. Actually, these analytical models cannot be applied directly for wear estimate since the thermomechanical loads near cutting edges keep changing with cutting conditions and tool wear evolution [24]. It is necessary to require the knowledge of these internal variables near cutting edges under different cutting conditions and their variation in tool wear evolution process. Therefore, some researchers have adopted experimental or analytical approaches to provide these inputs of internal variables as a function of cutting parameters and tool wear geometry [25,26]. However, in terms of difficulty of modeling a continuously progressive wear process and ever-changing process variables in predicting tool wear evolution, the popularity of this analytical modeling approach is also limited.

With rapidly increasing computer power and continually developing numerical methods, some attempts were made to numerically simulate cutting process, to calculate the thermo-mechanical loading histories and other process variables during cutting and in conjunction with analytical wear model to arrive at tool wear evolution at the cutting edges with reasonable accuracy. Therefore, some simulation has been implemented to reproduce tool wear evolution by integrating the above analytical wear models and cutting numerical models. The pioneering research of Xie [27] developed a 2D FE-based tool wear procedure based on the Python user program to predict the progression of tool wear. During each calculation cycle (time increment) of tool wear, FE analysis of chip formation and heat conduction during steady-state cutting was made to extract the process variables information essential for the inputs of Usui abrasive wear analytical model. According to the calculated wear rate, the tool nodal displacement in each time increment was obtained and hence the worn geometry can be updated based on nodal displacement. The next FE analysis cycle for predicting tool wear continued in an updated worn geometry until a use-defined tool reshape criterion was reached. Attanasio [28] extended the 3D FE simulation of tool wear by integrating the abrasive wear model proposed by Usui [22] and the high-temp diffusion wear model of Takeyama [21] to achieve a satisfactory wear estimate for 3D turning. Although some work has successfully reproduced the tool wear morphology in the experimental tests using the developed numerical models based description of different mechanical and physical wear mechanisms, 3D simulation of diamond physicochemical wear regarding the graphitization involving transformation of diamond into graphite and diffusion of newly-formed graphite into the workpiece has not yet been reported in metal cutting, especially for drilling. What’s noteworthy about modelling diamond physicochemical wear is that several researchers have attempted to mimic the transformation of tetrahedral diamond into hexagonal close-packing graphite and subsequent diffusion of graphite into transition metals and their alloys and find out suitable crystal orientations resistant to graphitization, using Molecular Dynamics method [29,30]. Unfortunately, Molecular Dynamics are used solely for simulating material removal process at nanoscale well within several hundred nanoseconds due to the combined limitations in available computer power, numerical methods and computational cost [12]. The aim of this paper is hence to analyze machining induced wear mechanisms underlying macroscale wear behavior and develop tool wear FE model based analytical description of wear mechanisms in diamond drilling of SiC_p_/Al composites. This was achieved by incorporating a combination of Usui wear model and graphitization induced chemical wear model into 3D FE model of diamond drilling as follows.

## 2. Experimental Work

### 2.1. Experimental Setup

**Figure 1:**
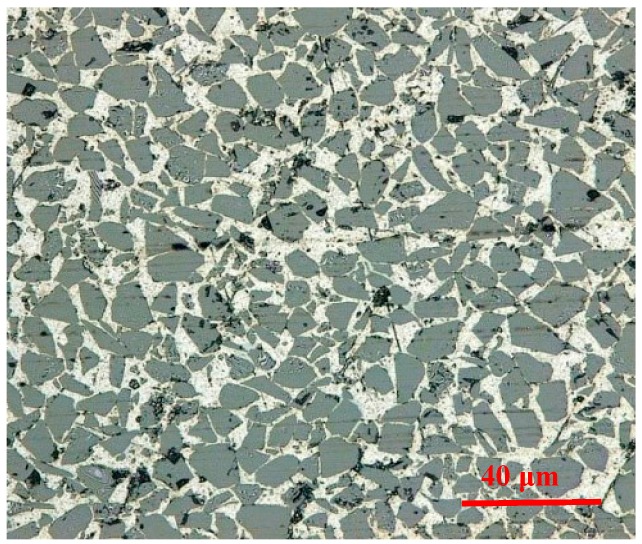
Micrograph of Al6063/SiC_p_/65p composites.

**Figure 2:**
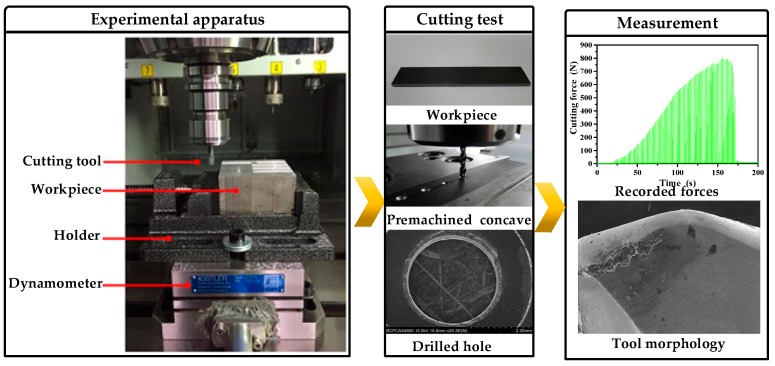
Experimental setup.

The material employed for the drilling tests was Al6063 matrix composites reinforced by 65% volume fraction SiC particulates (Al6063/SiC_p_/65p composites) fabricated through vacuum infiltration method. Figure 1 shows the microstructure of Al6063/SiC_p_/65p composites, in which SiC particulates are homogeneously distributed and not obviously clustered in the aluminum matrix. Its chemical composition determined using Energy-dispersive X-ray spectroscopy is given in Table 1. The drilling tests were performed on a DMU 80 monoblock machining center, equipped with a rotating 4-Component Dynamometer Kistler 9123 (Kistler Instrument China Ltd., Shanghai, China), as is shown in Figure 2. The wear morphologies of cutting tools were examined using 3D Laser Scanning Microscope VK-X200 (Keyence, Osaka, Japan). The measurement of drilling forces in machining was implemented using Kistler 9123. In this paper, the brazed polycrystalline diamond (PCD) drill and chemical vapor deposition (CVD) diamond coated drill were used to perform the cutting of Al6063/SiC_p_/65p composites. A summary of the experimental details upon tooling, workpiece and drilling conditions are given in Table 2. 

### 2.2. Wear Mechanisms

**Figure 3:**
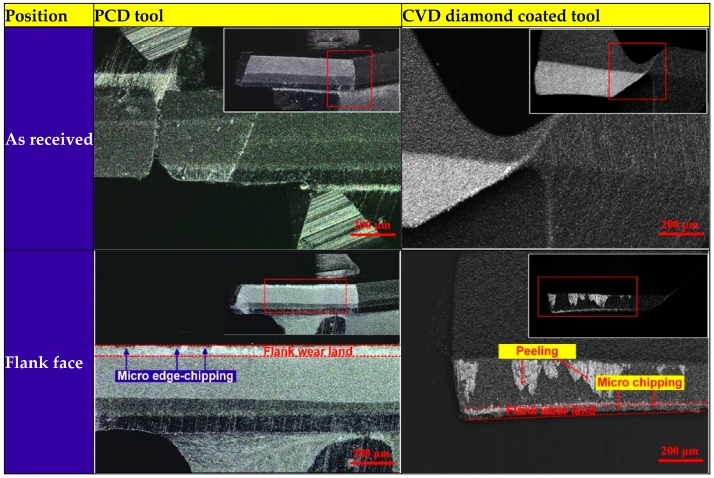

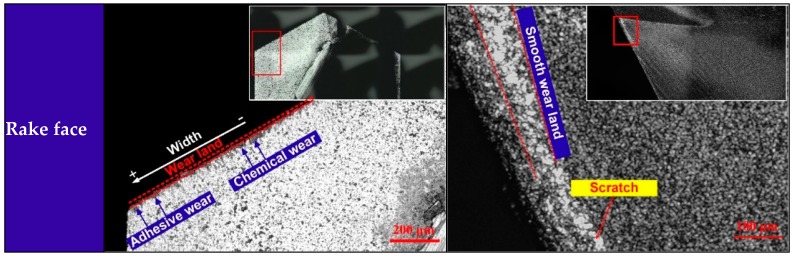
Wear morphologies of PCD and CVD diamond coated tools after drilling Al6063/SiC_p_/65p composites using 3D Laser Scanning Microscope VK-X200 (Keyence, Osaka, Japan).

Figure 3 shows the experimental findings of tool wear morphologies after drilling 10th hole using PCD and CVD diamond coated drills. The wear land was formed on the flank faces of PCD and CVD drill bits. The grooves and scratches parallel to cutting direction indicate the tool flank face suffered from severe abrasive wear. The combined action of two-body and multi-body abrasion at the tool-workpiece-chip interface resulted in the formation of flank wear land. The considerable and uneven wear morphologies on the rake face away from cutting lips of PCD drill and irregular wear land on the rake face near cutting lips was suspected to be caused by physicochemical wear.

Nevertheless, it is well-known that diamond tools cannot be used for cutting effectively transition metals and their alloys due to severe chemical wear. Actually, diamond tools can be used for cutting Cu and its alloys, although Cu is transition mental. And the significant graphitization of diamond tool has not been reported during cutting Copper and its alloys. The phase transformation of diamond into graphite during drilling of Al6063/SiC_p_/65p composites containing Cu is correlated to machining induced thermodynamics conditions with the catalysis of Cu in air atmosphere. The reciprocating actions of thin graphite layer formed by the oxidation of hydrogen chemisorbed on diamond surface with copper oxides and SiC particulates’ high-frequency scrape on newly-formed graphite film lead to the continual and significant occurrence of diamond graphitization [31]. More details of diamond tools wear during machining of SiC_p_/Al composites containing Cu is provided in [31]. It is found experimentally that PCD tool suffered from micro chipping, abrasive wear, adhesive wear and chemical wear, whereas CVD diamond coating tools suffered from abrasive wear, adhesive wear, delamination wear (peeling) and chemical wear. Tool wear in cutting is a complicated evolution process which is not formed by a unique wear mechanism but a combined action of mechanical, physical and chemical wear mechanisms [32,33]. In view of the complexity of tool wear mechanisms and theories, it is almost impossible to implement an overall wear model that considers all wear mechanisms involved in cutting to mimic tool wear progression. A simple but practical way to estimate tool wear is to only take into account those predominant wear mechanisms that occur under certain cutting conditions. The mechanically-induced abrasion and thermodynamically-activated graphitization are the predominant and common wear mechanisms for PCD and CVD tools during drilling Al6063/SiC_p_/65p composites containing Cu. 

## 3. Results

**Figure 4:**
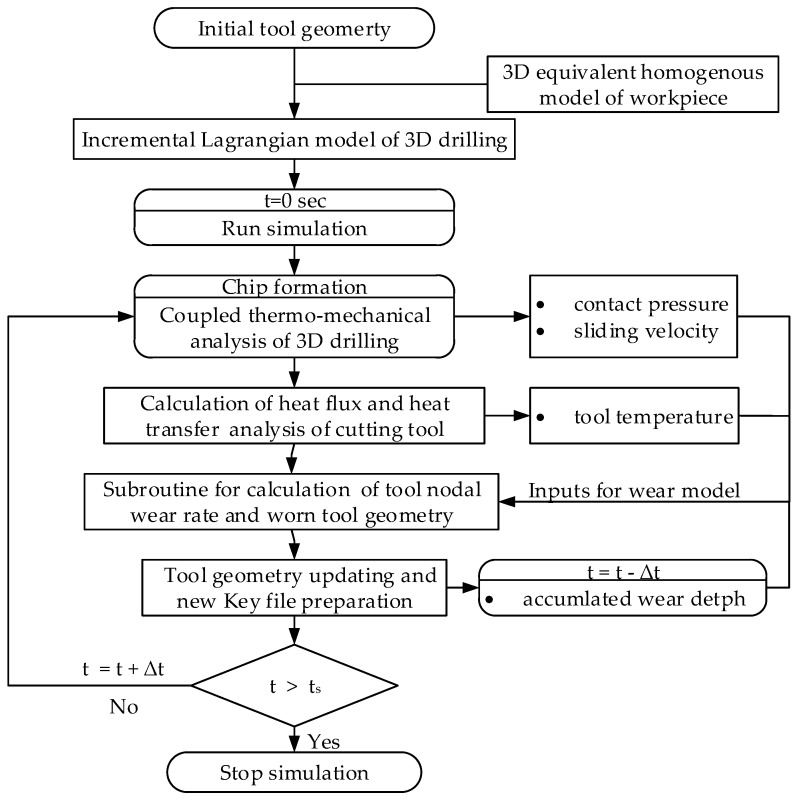
Flowchart of tool wear calculation procedure using FEM.

**Figure 5:**
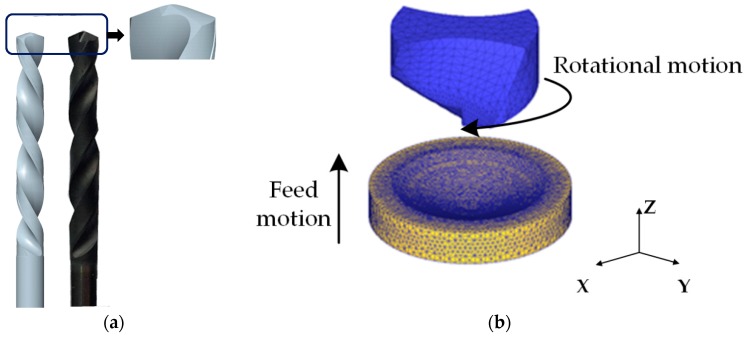
Drill modelling and simulation: (**a**) drill simplification; (**b**) FE modelling of drilling.

Figure 4 shows the flowchart of tool wear calculation procedure using DEFORM 3D FE codes. A 3D incremental Lagrangian drilling model—in which initial tool geometry and an equivalent homogenous material (EHM) based workpiece were incorporated—was established to implement an EHM drilling simulation of SiC_p_/Al6063 composites. The elasto-plastic workpiece and rigid tool are considered in the Lagrangian drilling model. To reduce computational cost, the modeling of drill bit is only focused on the tool tip part involving realistic drilling and the sharp edges is considered owing to newly-received drill bit employed at the beginning of drilling (Figure 5a). The motions for tool feed and rotation are imposed on the tool center axis. For workpiece modelling, the workpiece part neighbored to the tool tip are accounted for in drilling model and to arrive at stable drilling as soon as possible, a cone-like concave machined surface is firstly pre-built on the workpiece surface, as shown in Figure 5b.

The chip formation and heat transfer analysis are implemented by running 3D coupled thermomechanical drilling model to provide the inputs of process variables (contact stress at the tool surface, relative sliding velocity between the chip and tool, temperature at the chip-tool interface) required for tool wear estimate and subsequent tool worn geometry updating. The distributions of these process variables were then input into a subroutine for tool wear calculation based on the analytical description of the dominant diamond wear mechanisms during drilling Al6063/SiC_p_/65p composites. According to the calculated tool nodal wear rate and worn geometry, the tool geometry in finite element codes is updated and new Key files are prepared for the next simulation cycle. 

### 3.1. Chip Formation

#### 3.1.1. Material Model

In modelling of forming, manufacturing and structural mechanics, the characterization of material thermomechanical behaviors is often made using a widely-used phenomenological constitutive model. The thermomechanical behaviors of SiC_p_/Al6063 composites during drilling were reflected by using a phenomenological constitutive model determined through quasi-static and dynamic compression tests. To simulate chip formation during drilling, a generalized Johnson-Cook plasticity model was adopted for describing the material responses of SiC_p_/Al6063 composites in cutting.

(1) $$\sigma =\left(A+B\varepsilon_{p}^{n}\right)\;\left[1+C\ln\left(\dot{\varepsilon }/{\dot{\varepsilon }}_{0}\right)\right]{\left(\dot{\varepsilon }/{\dot{\varepsilon }}_{0}\right)}^{\alpha }\left[D-E{\left(T^{*}\right)}^{m}\right]$$

With

(2) $$T^{*}=\{\begin{array}{c} 0;T<T_{\mathrm{room}}\\ \left(T-T_{\mathrm{room}}\right)/\left(T_{\mathrm{melt}}-T_{\mathrm{room}}\right);\;T_{\mathrm{room}}\leq T\leq T_{\mathrm{melt}}\\ 1;T>T_{\mathrm{melt}} \end{array}$$

(3) $$D=D_{0}\exp\left[k{\left(T-T_{b}\right)}^{\beta }\right]$$

where A, B, C, n, α, m, E, $D_{0}$, k, β are material coefficients, σ and $\varepsilon_{p}$ are respectively flow stress and effective plastic strain, ${\dot{\varepsilon }}_{0}$ is the reference strain rate, $T_{\mathrm{melt}}$ is melting temperature, T workpice temperature and $T_{\mathrm{room}}$ room temperature, $T_{b}$ reference temperature. 

**Figure 6:**
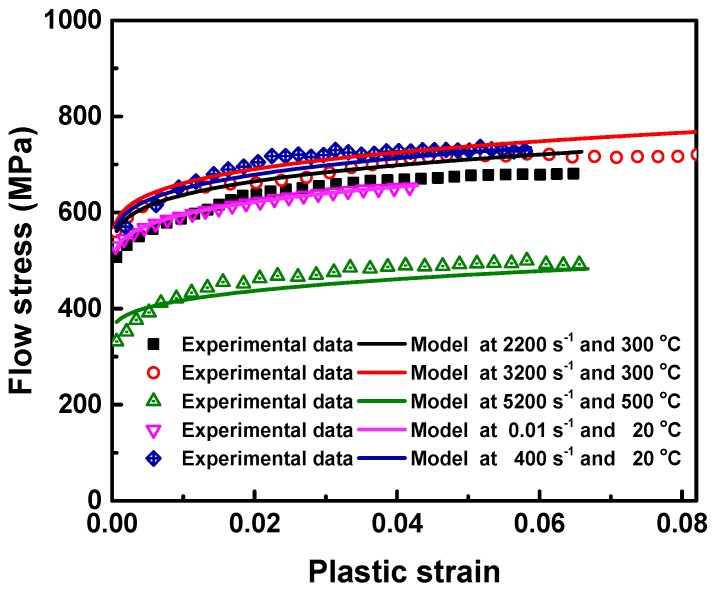
Comparison of the experimental data and prediction results of the identified model through multi-objective strategy.

A multi-objective method for model parameters identification proposed in [34] was employed to find a feasible set of parameter estimates so that the formulated plasticity model by these parameters can capture the material behaviors in both quasi-static and dynamic loading modes equally well. Using multi-objective identification strategy, the obtained values of material parameters of the generalized Johnson-Cook plasticity model for Al6063/SiC_p_/65 composites are given in Table 3. The overall fit standard error and R^2^ is respectively 18.048 MPa and 97.92%. The good agreement of model prediction with test data under different loading conditions is illustrated in Figure 6. Apart from material parameters for J-C plasticity model, the physical and mechanical properties of Al6063/SiC_p_/65 composites are presented in Table 4.

#### 3.1.2. Chip Separation

Chip separation during chip formation is correlated to material failure mechanisms. Generally, the failure mechanisms of particulate reinforced metal matrix composites (PRMMCs) are manifested in: (i) particulate cracking, (ii) debonding interface between particulate and metal matrix and (iii) nucleation, growth and coalescence of voids within metal matrix [35]. Which of the three mechanisms dominated in composites failure is dependent on microstructural morphology information of particulate volume fraction, shape, size, spatial location and on mechanical behavior of metal matrix.

**Figure 7:**
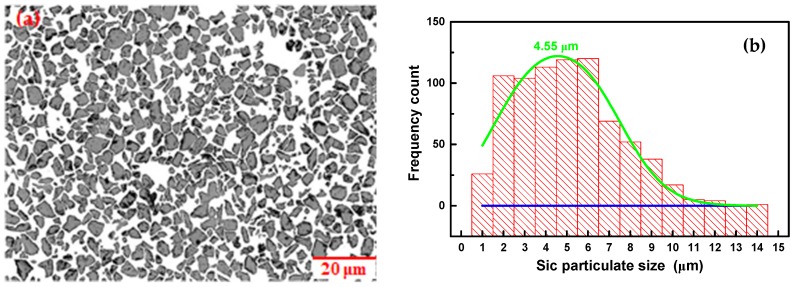
(**a**) Microstructure and (**b**) SiC particulate size distribution of Al6063/SiC_p_/65p composites.

**Figure 8:**
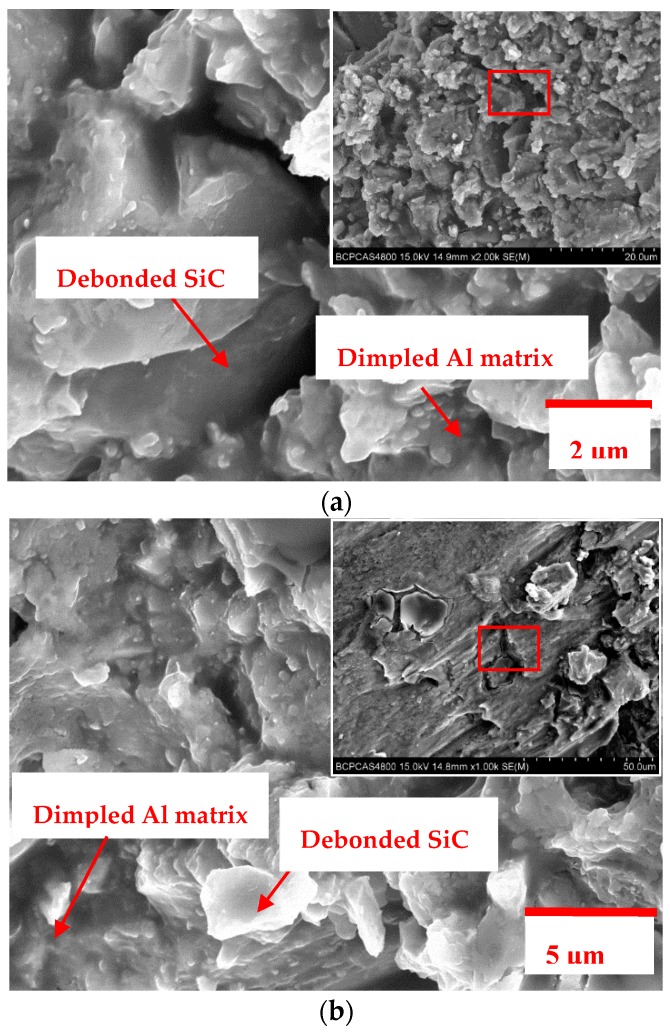
Fracture morphologies of Al6063/SiC_p_/65p composites after (**a**) uniaxial compression and (**b**) machining.

The experimental findings of Lloyd [36] indicate that only when particulate size is ≥20 μm, particulate cracking becomes the dominant failure mode in PRMMCs. This is also confirmed in Xie [37] on the investigation of defect formation mechanism in machining SiC_p_/Al composites using multi-phase FE model. Provided that reinforcement particulates are distributed uniformly and have no preferred orientation in metal matrix, the area fraction of reinforcement particulates on any cross section with large enough area should be approximately equal to the volume fraction of reinforcement particulates. This paper employed the statistical characterization methodology of PRMMCs proposed by Zhang [38] to quantify the microstructural information and estimated the mean effective diameter of SiC particulates to be about 4.55 μm according to the microstructure containing large numbers of SiC particulates shown in Figure 7. Therefore, the damage and failure for Al6063/SiC_p_ composites is accompanied by the nucleation of microvoids originated from matrix cracking and particulate/matrix interface debonding, growth and coalescence of voids. The fracture morphologies of Al6063/SiC_p_/65p composites after uniaxial compression and machining in Figure 8 are also shown to be made up of dimpled Al matrix, debonded SiC particulates and fewer cracked SiC particulates. As can be seen, the occurrence of damage and failure for Al6063/SiC_p_/65p composites is of localized characteristics and the damage and failure modes involve Al matrix cracking and interface degradation induced matrix fracture around large numbers of particulate inclusions.

The Cockroft & Latham damage criterion is shown to be capable of incorporating the tensile stress effect on chip formation during drilling [39,40]. Hence, the Cockroft & Latham damage model is employed for determining material damage and resultant chip segmentation during drilling Al6063/SiC_p_/65p composites.

(4) $$D=\overset{\varepsilon_{f}}{\underset{0}{\int }}\sigma \left(\frac{\sigma^{*}}{\sigma }\right)d\varepsilon_{p}$$

where D is the damage state variable for characterizing continuum damage softening. And when D reaches the critical value $D_{cr}$, the chip separation is triggered by the corresponding elements deletion. $\sigma^{*}$ the maximum principal stress.

### 3.2. Heat Generation

**Figure 9:**
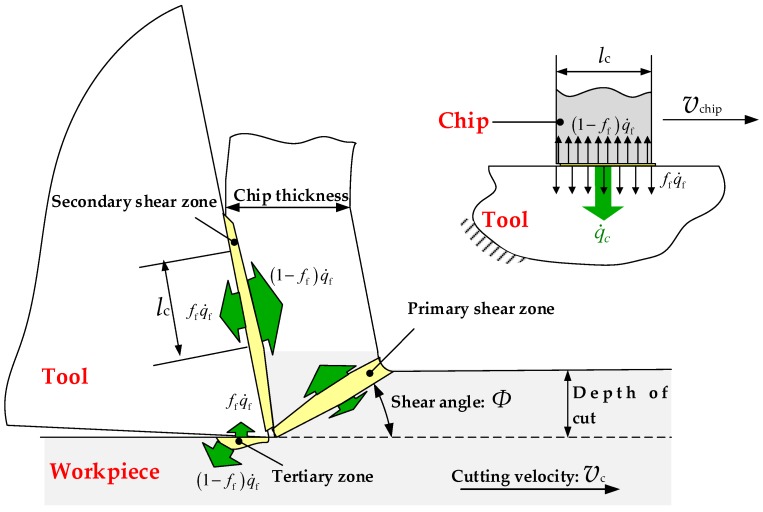
Schematic of heat partitioning in the chip formation process.

The temperature during cutting process plays a major role in tool wear evolution and wear mechanisms [41]. The heat generation during machining is divided into plastic deformed heat and friction induced heat. Figure 9 shows the schematic of heat partitioning in the chip formation process. The converted heat rate ${\dot{q}}_{p}$ by plastic deformation leads to the workpiece temperature variation ΔT in material forming and machining.

(5) $${\dot{q}}_{p}=\eta_{p}\tau_{\Phi }d\gamma =\rho C_{p}\Delta T$$

where $\eta_{p}$ is Taylor-Quinney coefficient that indicates the fraction of plastic work conversion into heat, $\tau_{\Phi }$ effective adiabatic shear flow stress, γ effective plastic shear strain, ρ and $C_{p}$ the workpiece density and specific heat, respectively.

The mechanical formulations for sticking and slipping contact along local tangent directions are respectively defined as shear friction and Coulomb friction in terms of the division of slipping ($\mu p\leq \tau_{\mathrm{crit}}$) and sticking ($\mu p>\tau_{\mathrm{crit}}$) regions in possible contact domain.

(6) $$\tau_{f}=\{\begin{array}{c} \mu p,\;\mu p\leq \tau_{\mathrm{crit}}\\ \tau_{\mathrm{crit}}=m\sigma /\sqrt{3},\;\mu p>\tau_{\mathrm{crit}} \end{array}$$

where $\tau_{\mathrm{crit}}$ is the critical shear flow stress, of which the magnitude is generally $\sqrt{3}$ times lower than the tensile yield stress σ, p the chip-tool interface pressure, μ the friction coefficient. The friction heat $q_{f}$ at the workpiece-tool interface can be computed according to Equations (6) and (7).

(7) $${\dot{q}}_{f}=\eta_{f}\int \tau_{f}dv_{s}$$

where $\eta_{f}$ is the converted fraction of friction work into heat, $k_{\mathrm{interface}}$ interface heat transfer coefficient. The amount of heat flux into the tool ${\dot{q}}_{f}^{\mathrm{tool}}$ and workpiece ${\dot{q}}_{f}^{\mathrm{work}}$ can be given by the following quantitative relations of heat partitioning at the tool-workpiece interface.

(8) $${\dot{q}}_{f}^{\mathrm{tool}}=f_{f}{\dot{q}}_{f}+{\dot{q}}_{c}$$

(9) $${\dot{q}}_{f}^{\mathrm{work}}=\left(1-f_{f}\right){\dot{q}}_{f}-{\dot{q}}_{c}$$

with

(10) $${\dot{q}}_{c}=-k_{\mathrm{int}}\left(T_{\mathrm{int}}^{\mathrm{tool}}-T_{\mathrm{int}}^{\mathrm{work}}\right)$$

where $f_{f}$ is the fraction of frictional heat flux $q_{f}$ transferred to tool, $k_{\mathrm{int}}$ the interface heat transfer coefficient, $T_{\mathrm{int}}^{\mathrm{tool}}$ and $T_{\mathrm{int}}^{\mathrm{work}}$ the tool and workpiece temperature near the workpiece-tool interface, respectively.

### 3.3. Diamond Wear Modelling

The rate of volume loss on the tool per unit area per unit time is calculated by a wear rate model considering the predominant wear mechanisms. Due to highly abrasive characteristics of SiC_p_/Al6063 composites, hence the abrasive model proposed by Usui was adopted to include the contact stress p, relative sliding velocity $v_{s}$ and tool temperature T dependencies. According to the analysis of wear mechanisms in Section 2.2, hence, the coupled abrasive-chemical wear model based description of the dominant wear mechanisms of diamond during drilling Al6063/SiC_p_/65p composites was implemented into a subroutine for tool wear estimate.

(11) $$\{\begin{array}{c} \frac{\partial W}{\partial t}=\frac{\partial W_{a}}{\partial t}=Apv_{s}\exp\left(-\frac{B}{T}\right)\;T\leq T_{\mathrm{trans}}\\ \frac{\partial W}{\partial t}=\frac{\partial W_{a}}{\partial t}+\frac{\partial W_{g}}{\partial t}=Apv_{s}\exp\left(-\frac{B}{T}\right)+Gp^{n}\exp\left(-\frac{E}{RT}\right)\;T>T_{\mathrm{trans}},p\leq p_{\mathrm{trans}} \end{array}$$

where ∂W/∂t is the overall tool wear rate, $\partial W_{a}/\partial t$ is the wear rate calculated according to Usui abrasive model. $\partial W_{g}/\partial t$ is the wear rate calculated according to graphitization induced chemical wear model that extends the Arrhenius law to include pressure-dependence, E activation energy, R gas constant, A, B, n and G experimentally calibrated coefficients. $T_{\mathrm{trans}}$ and $p_{\mathrm{trans}}$ are the activation temperature and pressure for the transformation of diamond into graphite (respectively equal to 500 °C and 15 GPa under Cu catalysis) [31].

In this work, the coupled thermomechanical FE model of 3D drilling was applied to drilling process simulation to obtain the distribution of the process variables such as the temperature at the workpiece-tool interface, the relative sliding velocity between the workpiece and tool, contact pressure on the tool surface during drilling. The combined abrasive-chemical wear model in Equation (11) was implemented into an appropriate subroutine for calculating the wear rate and wear variables at the tool nodes in contact with the deforming workpiece by using the available information about thermo-mechanical variables, nodal area and time step. According to the estimated wear rate through the subroutine, the nodal displacement on the tool surface at current time step was computed. The tool geometry was recalculated and updated based on the computed nodal displacement and updated tool. Then a second wear estimate at next time step started with the update worn tool geometry. As this process was repeated, the tool wear evolution was reproduced throughout the machining process.

## 4. Results and Discussions

### 4.1. Cutting Forces

**Figure 10:**
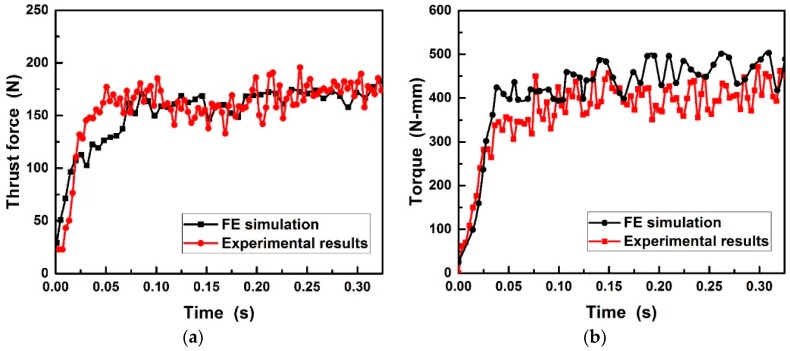
Comparison of (**a**) thrust force and (**b**) torque between FE simulation and experiment during drilling Al6063/SiC_p_/65p composites using brazed PCD drill.

**Figure 11:**
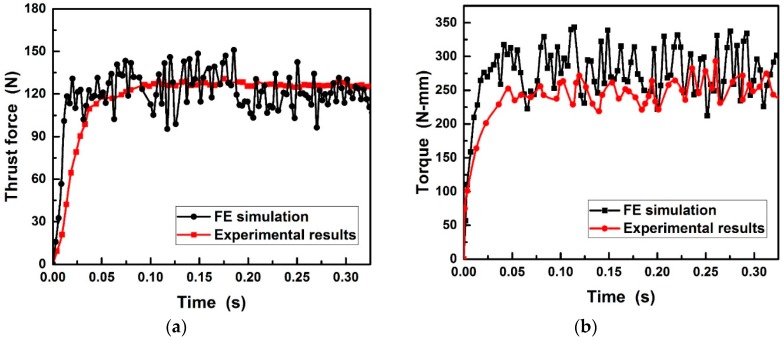
Comparison of (**a**) thrust force and (**b**) torque between FE simulation and drilling experiment performed during drilling Al6063/SiC_p_/65p composites using CVD diamond coated drill.

Based on the experimental details in Table 2, the simulated and experimental cutting forces during drilling Al6063/SiC_p_/65p composites using PCD and CVD drills is presented in Figure 10 and Figure 11. It can be found that the good agreement between simulated and experimental in thrust force is reached except at the beginning stage of drilling. However, the 14.4% and 10.9% overestimation for the torque acting on PCD and CVD diamond coated drills may be partly due to the small difference between realistic and simulated tools geometry to which the torque is more sensitive and partly due to the restricted material flow by the fixed lateral wall of the workpiece. As evident, the developed drilling model allowed a fairly accurate prediction of the cutting forces, while it overestimates the torque that has less influence on tool wear. As seen from Figure 10 and Figure 11, under the same cutting conditions, the cutting forces applied on PCD drill was higher than those on CVD diamond coated drill. The increase in cutting forces could result in easy damage and consequent breaking of the chips.

### 4.2. Chip Morphology

**Figure 12:**
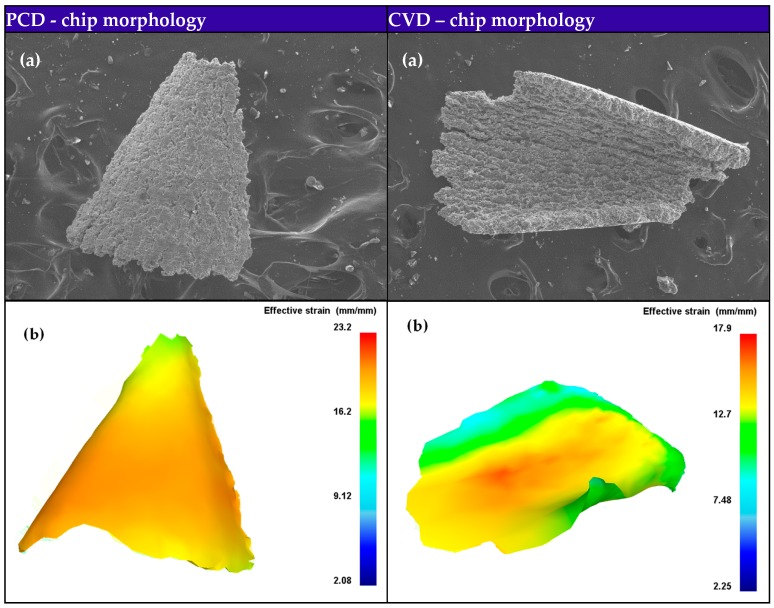
(**a**) Experimental and (**b**) simulated morphologies of the chips formed using PCD and CVD drills.

**Figure 13:**
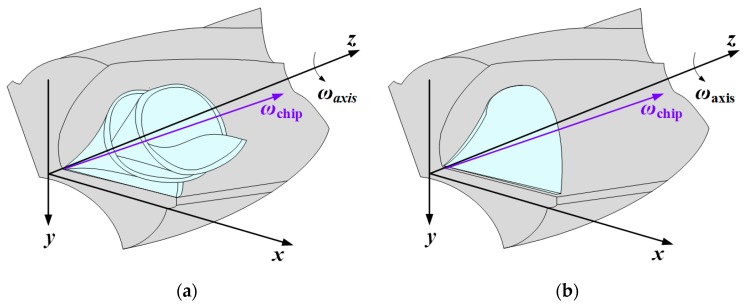
Schematic of chip formation in drilling of (**a**) Al alloy and (**b**) SiC_p_/Al composites.

The disclosure of chip formation mechanism contributes to assisting the redesign of tool geometry and optimizing the machining processes. Comparison of the experimental and simulated morphologies of the chips formed using PCD and CVD diamond coated drills is depicted in Figure 12. The simulated chip shape matched well with experimental obtained chip morphology, especially in chip curling. Additionally, the chip produced by straight cutting lip of PCD drill is more discontinuous and fragmentary than that by curved cutting lip of CVD diamond coated drill during drilling Al6063/SiC_p_/65p composites. As show in Figure 13a, when drilling the corresponding Al alloy, the force originated from chip formation drives the chip moving up along the flute and rotating by its own chip axis $\omega_{\mathrm{chip}}$, simultaneously the force normal to the flute surface from the drill flute promotes the chip curling by twisting the chip and eventually leads to spiral chip formation during drilling Al alloy [42]. Whereas, Al6063/SiC_p_/65p composites, having high volume fraction of SiC reinforcement and consequently low ductility and resistance to bending, generated small fragmentary chips (Figure 13b). The apparent difference of chip morphology during drilling requires in-depth understanding of deformation and failure mechanisms at microstructure scale of Al alloy and SiC_p_/Al composites subjected to shear loading that lead to different chip formation.

**Figure 14:**
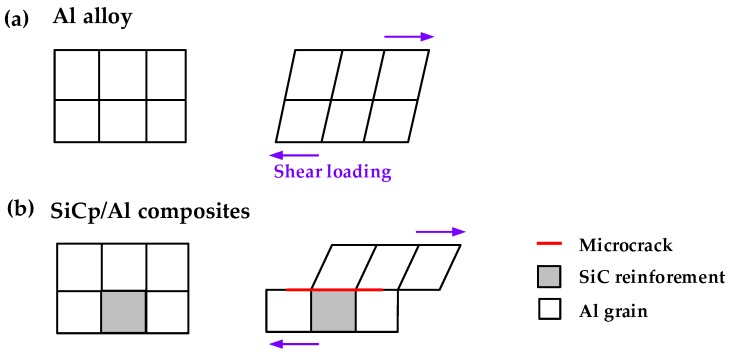
Deformation and failure mechanisms at microstructure scale of (**a**) Al alloy and (**b**) SiC_p_/Al composites when subjected to shear loading.

**Figure 15:**
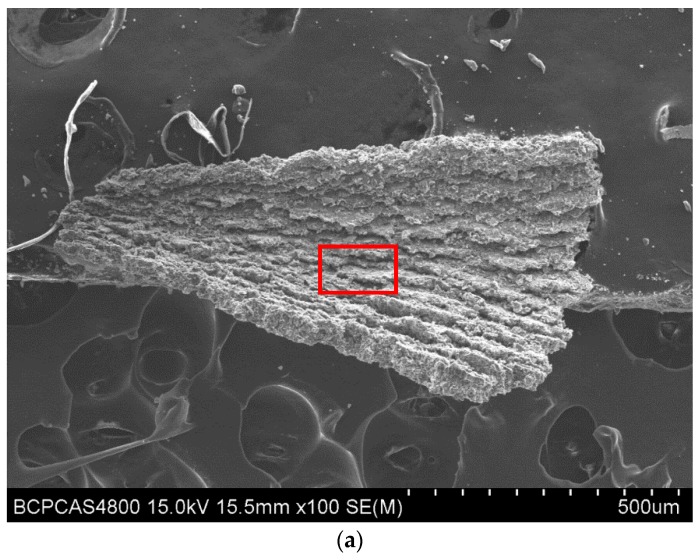

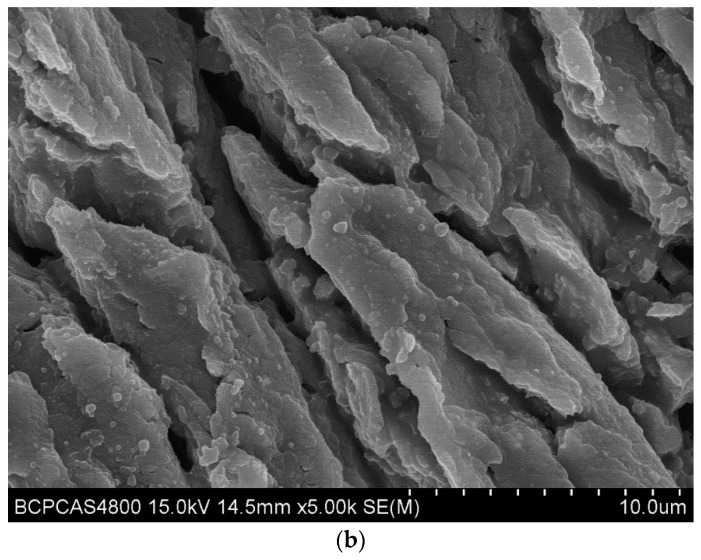
SEM micrograph of the chip: (**a**) free surface, (**b**) image magnification of rectangle region in (**a**).

As can be seen from Figure 14, unlike coordinated deformation of Al grains in drilling Al alloy, when drilling Al6063/SiC_p_/65p composites, the localized shear applied by cutting lips would cause the deformation incompatibility of SiC particulates and Al grains in the primary shear zone, thus making the microcracks initiated at the interface of SiC particulates and the Al matrix due to uncoordinated deformation. Simultaneously, the machining induced compressive stress relief in the freshly generated chip would lead to the formation of a microcracked region with numerous discontinuous cracks in the chip free surface due to the presence of high volume faction brittle phase SiC in Al6063/SiC_p_/65p composites (Figure 15). As the drill travels, the newly-formed chip slides outward along the shear plane, meanwhile the microcracked region on the chip free surface propagates further along the rake face. When the chips gradually bend to some extent in which the propagation of the crack to the cutting lip due to microcrack coalescence, sudden brittle fracture of the chip would occur. Therefore, the formed chip when drilling Al matrix composites reinforced by SiC particulates is easily broken and fragmented. Additionally, the chip formation is found to be associated with drill geometry, especially drill flute. Often, the more freely the chip moves, the larger the formed chip length is. When the chip comes into the drill flute, the chip motion is impeded by the flute. The flat flute face of PCD drill would restrict the chip curling, while the curved flute face of CVD diamond coated drill can accommodate the curling deformation of the chip. Moreover, compared to the straight lips of PCD drill, the curved lips of CVD diamond coated drill result in larger uncut chip thickness and increased bending strength of the resultant chips, thereby delaying chip-breaking. Therefore, the chip produced using CVD diamond coated drill is longer and bent more severely, compared to that using PCD drill.

### 4.3. Tool Temperature

**Figure 16:**
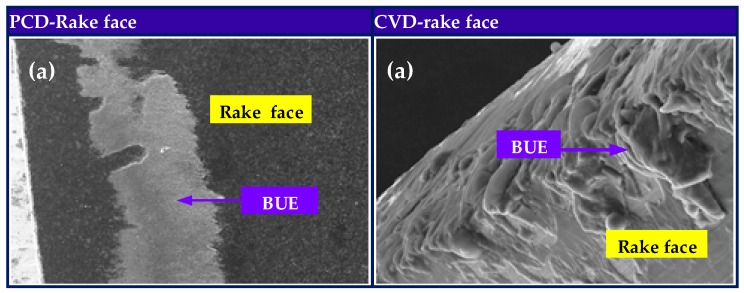

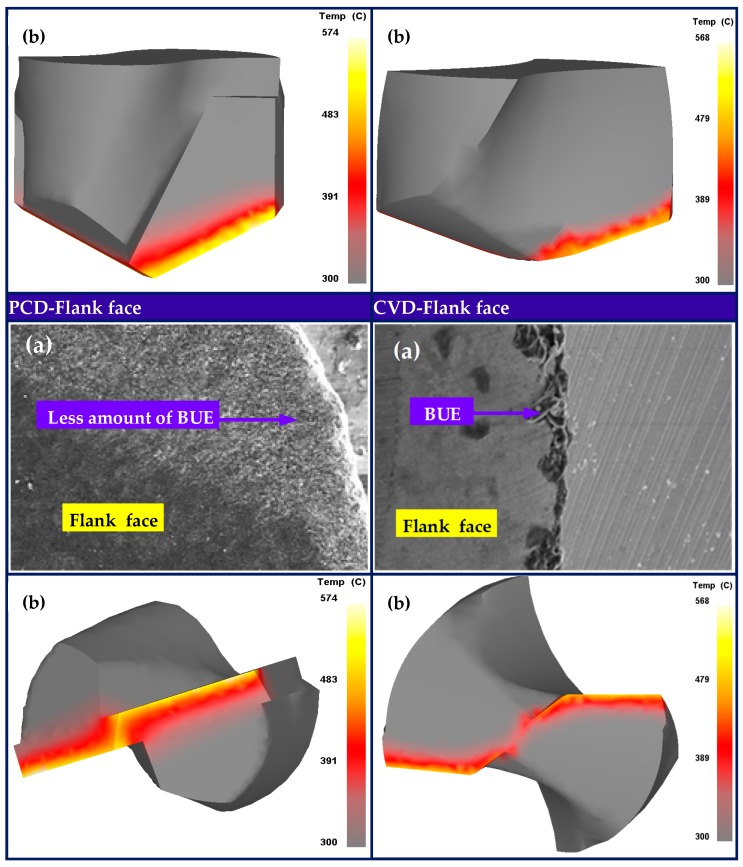
(**a**) Built-up edge and (**b**) temperature distribution on rake and flank faces of PCD and CVD diamond coated drills.

Increase of the friction force at the tool-chip interface caused by heat localization during high-speed cutting leads to heat localization and high temperature rise in the cutting zone. Consequently, the built-up edge (BUE) often occurs by adhering to the cutting edges of drill bit and this adhesion on the drill surfaces would reduce the hole surface quality and result in the adhesive wear on the cutting edges by intermittent growth and scraping off of BUE. Due to semi-enclosed characteristics of drilling operation, the temperature distribution of drill surface is difficult to measure, especially on the cutting edges. However, a more important issue in tool wear estimate is temperature prediction. To some extent, the distribution of BUE on the drill bit can provide some valuable information and reference about temperature distribution. In Figure 16b, larger high-temperature affected zone is formed on the rake face of PCD drill compared to that of CVD diamond coated drill and thus the larger active area of BUE on the PCD drill than on the CVD diamond coated drill is observed, as shown in Figure 16a. BUE is not observed near the PCD cutting lips, while there exists BUE on the drill faces far away cutting lips. This implies that the BUE formed near cutting lips were scraped off soon due to high-frequency friction effect from SiC particulate. Again, the agreement of experimentally determined BUE distribution on PCD and CVD diamond coated drills bit with numerically simulated high-temperature affected zone instills confidence in the developed 3D drilling FE model for tool wear progression.

### 4.4. Tool Wear

**Figure 17:**
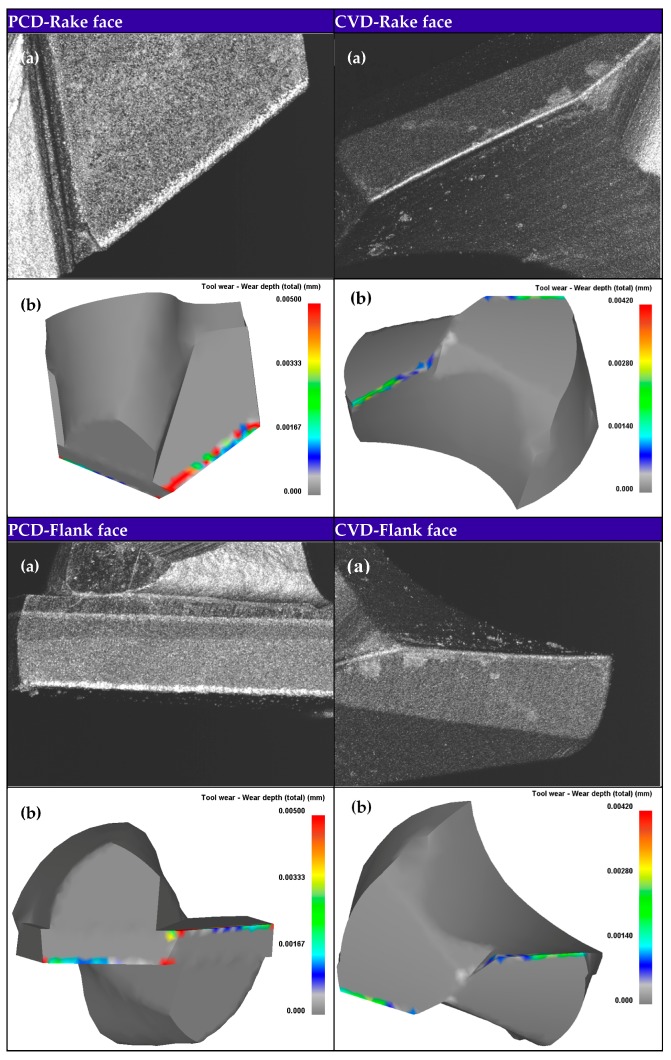

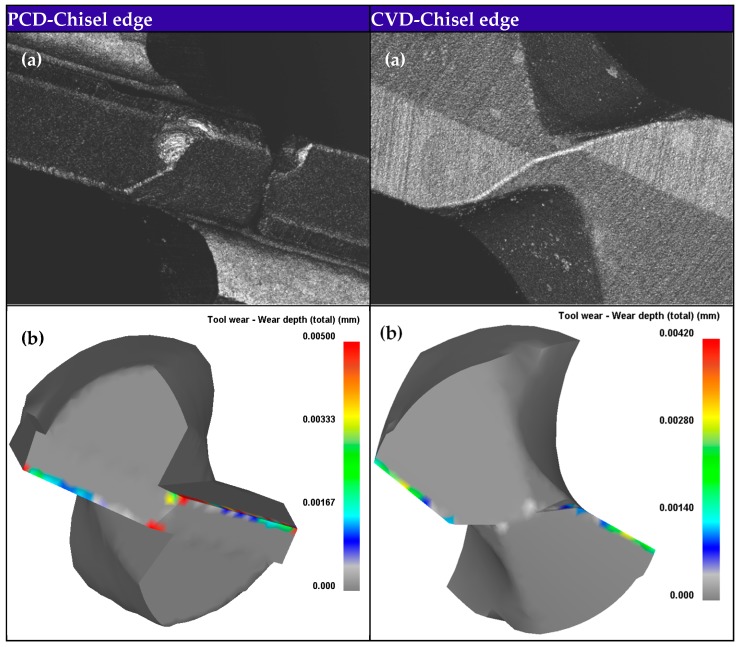
(**a**) Experimental and (**b**) simulated wear morphologies of PCD and CVD drills after drilling a hole.

Based on the relative velocity between the drill and chip, the temperature at the drill-chip interface and contact stresses at the drill surface obtained through the coupled thermomechanical analysis of chip formation during 3D drilling, the subroutine for calculating combined abrasive-graphitization wear rate on tool nodes is called to estimate tool wear and update tool geometry. The tool wear calculation cycle is continually repeated until a drilled hole is accomplished. In Figure 17, the experimentally observed and numerically simulated wear morphologies of PCD and CVD diamond coated drills are compared after drilling a hole. As can be seen, the presence of graphitization induced chemical wear resulted in the irregular evolution of the simulated tool wear showing a good agreement with experimental observations, compared to even wear morphology caused by only abrasive wear. PCD drill with straight cutting lips suffered form more severe wear than CVD diamond coated drill with curved cutting lips in drilling Al6063/SiC_p_/65p composites, especially in chisel edge. Table 5 shows the comparison of the maximum flank wear width (MFWW) between experiment and FE simulation. The similar wear morphologies and matched MWFW of both PCD and CVD diamond coated drills between experimental results and model prediction validate the feasibility and reliability of the developed 3D tool wear FE model based analytical description of the dominant diamond wear mechanisms taking into account the abrasion and graphitization.

## 5. Conclusions

In this paper, an attempt was made to predict the wear evolution of diamond tools during drilling SiC_p_/Al composites by using the developed drilling FE model based analytical description of the dominant diamond wear mechanisms. The diamond tool wear in drilling SiC_p_/ Al6061 composites is mainly attributed to the combined effects of abrasion from SiC particulates and graphitization catalyzed by Cu in the Al6061 matrix. The combined abrasive-chemical wear model coupling Usui abrasive model and Arrhenius extended graphitization wear model was implemented by a user-defined subroutine for the tool wear rate estimate that is formulated by some process variables such as the relative velocity between the drill and chip, the temperature at the drill-chip interface and contact stresses at the drill surface. These process variables can be available from the coupled thermomechanical FE analysis of 3D drilling. The developed FE model for tool wear estimate was validated for feasibility and reliability by comparing numerically simulated tool wear morphology and experimentally observed results after drilling a hole using PCD and CVD diamond coated drills. The similar cutting forces, chip and tool wear morphologies between experimental results and model prediction indicate that the developed 3D drilling FE model, combined with a subroutine for tool wear estimate can provide good prediction not only in cutting forces and chip shape but also in tool wear behavior. The presence of graphitization induced chemical wear resulted in the irregular evolution of the simulated tool wear, which demonstrates a good agreement with experimentally observed tool wear. Hence, it is possible to utilize the tool wear FE simulation based analytical description of the dominant wear mechanisms to estimate the overall tool wear.

The developed diamond tool wear FE model can be feasibly extended to the investigation of diamond tool wear evolution with various diamond tool geometries in cutting different workpiece materials once calibrated the tool wear model and determined the workpiece constitutive model. Capability of evaluating the dominant diamond wear mechanisms effects on tool wear contributes to the redesign of tool geometry and optimization of cutting process.

## Figures and Tables

**Table 1:** Chemical composition of Al6063/SiC_p_/65p composites.

Element	Al	Mg	Cu	Si	C	Others
wt %	38.33	0.48	1.51	51.24	8.43	margin

**Table 2:** Summary of experiment details.

Items	Contents	
Tooling		
Tool Manufacturer	Zhengzuan Precision Manufacture Co., Ltd. (Zhengzhou, China)	
Drill bit material	PCD	CVD diamond
Diameter *d* (mm)	3	3
Point angle ϕ (°)	120	140
Rake angle γ (°)	0	
Relief angle α (°)	10	
Helix angle ω (°)	30	30
**Workpiece**		
Material	Al6063/SiC_p_/65p composites	
Thickness (mm)	2	
**Cutting Conditions**		
Operation	Drilling	
Rotational speed n (rpm)	2000	
Feed velocity $v_{f}$ (mm/min)	100	
Drilling environment	Dry	

**Table 3:** Material constants for generalized J-C plasticity model for Al6063/SiC_p_/65p composites.

*A*/MPa	*B*/MPa	*C*	*D*_0_	*E*	n	m	*α*	*β*	*k*	*T*_b_
501	449	0.0002	0.291	0.8995	0.2539	1.602	0.0105	0.1675	0.4781	98.2

**Table 4:** Physical and mechanical properties of Al6063/SiC_p_/65p composites [34].

Notation	Material Properties	Value
$\rho_{c}$	Density (kg/m^3^)	2960
$C_{p}$	Specific heat capacity (J/kg)	750
*α*	Coefficient of thermal expansion (10^−6^)	7.7
*κ*	Thermal conductivity (W/m∙)	175
E	Elastic modulus (GPa)	221
*υ*	Poisson’s ratio	0.21
$T_{\mathrm{room}}$	Room temperature	20
$T_{\mathrm{melt}}$	Melting point	635
${\dot{\varepsilon }}_{0}$	Reference strain rate	0.01
*η*	Inelastic heat fraction	0.9

**Table 5:** Comparison of the MFWW between experiment and FE simulation.

Cutting Tool	Experimental MFWW	Simulated MFWW	Relative Error
PCD	43.237 μm	47.544 μm	9.96%
CVD	38.119	37.608 μm	1.34%
